# On the use of a Modified Kubler-Ross Model of Grief to Treat Bereavement in Schizophrenia

**DOI:** 10.7759/cureus.12041

**Published:** 2020-12-12

**Authors:** Bolaji Yoade, Nkolika Odenigbo, Jaquana Jones, Jisha Kallikkadan, Ayodeji Jolayemi

**Affiliations:** 1 Psychiatry, Interfaith Medical Center, Brooklyn, USA; 2 Psychiatry, American University of Antigua College of Medicine, Coolidge, ATG

**Keywords:** schizophrenia, bereavement, cognitive behavioral therapy, grief

## Abstract

Models and therapeutic approaches to bereavement have focused on patients without mental illness, with limited studies done on patients with psychiatric disorders, specifically schizophrenia. A question arises as to how the models of bereavement may be modified in schizophrenia and what are the possible adjustments in bereavement counseling for those with schizophrenia. We describe the case of a 50-year-old African American male with a history of schizophrenia. He was admitted to the psychiatric inpatient service after he was found living at home with the decomposing body of his dead mother for several days. Positive and Negative Syndrome Scale (PANSS) score was 32 on the positive scale and 39 on the negative scale at the beginning of his hospital course. A modified model of bereavement was formulated in light of his acute psychotic symptoms, based on Kubler-Ross and Cognitive theory, which consisted of 20 sessions implemented over four weeks. Initial sessions were supportive and focused on establishing rapport, psychoeducation about the concept of dying, and losing support systems. Later sessions focused on the exploration of cognitive beliefs and targeting cognitive distortions. By the end of the fourth week, the patient did not seem to exhibit delusions and more readily accepted the finality of his mother's death. PANSS score was 8 on the positive scale and 19 on the negative scale by the end of his hospital course. We utilized modified Kubler-Ross and bereavement counseling models in this patient with a resolution of the psychotic denial phase of his loss. Further studies need to be done on the possible utility of our modified model and modified therapeutic approach for bereavement in patients with schizophrenia.

## Introduction

Elizabeth Kubler-Ross proposed the five stages of grief describing the experience people may have after a loss. Denial, anger, bargaining, depression, and acceptance are the stages proposed by Ross as tools to help us frame and identify what people may be experiencing at the time of grief. These stages are widely known as the Kubler-Ross stages of grief and were characterized based on studies on patients without mental illness [1]. It has also formed the basis of psychotherapeutic approaches to grieving individuals [2]. Limited studies on bereavement have been done on patients with mental illness. Current literature on bereavement in schizophrenia serves to focus on the mourning experiences of caregivers of the patients and how those losses may lead to a pattern of chronic grief for the remaining caregivers themselves [3]. Other literature focuses on the experiences of mourning of the former self and the coming to terms with the initial diagnosis of schizophrenia [4]. However, there has been limited application of therapeutic approaches to bereavement in patients with mental illness. We present a case of a patient with schizophrenia who was treated with 20 sessions of modified cognitive behavioral therapy to allow him to move through a modified Kubler-Ross model of bereavement.

## Case presentation

We present a case of a 50-year-old African American male with a past psychiatric history of schizophrenia who was admitted to our ED after his neighbors complained of a foul smell coming from his apartment. The patient presented with mostly negative symptoms of schizophrenia. He initially stated that his neighbors called the police because “the weather is wet” and his allergies were out of control. He was found at home with his deceased mother who had shown signs of decomposing. When he was initially assessed, he only complained of feeling short of breath due to a foul smell of tuna fish coming from around his mother. When asked about his deceased mother he stated she was watching television and that she still had a pulse. According to the patient he had been giving his mother tea and medication for the past couple of days and had recently given her aspirin for her headaches before police arrival. It was determined that he needed psychiatric evaluation and he was brought into the ED by emergency medical services. He was determined to be acutely psychotic with poor reality testing and insight likely due to poor medication compliance. He continued to remain unaware of his mother's death during evaluation. He was admitted to inpatient psychiatry service for stabilization. On initial evaluation in inpatient psychiatry, the patient continued to maintain that he could communicate with his mother and spoke to her with a red phone next to his hospital bed. He was withdrawn, disheveled, malodorous, blunted in affect, internally preoccupied, and intermittently responding to internal stimuli (Table 1). PANSS score was 32 on the positive scale and 39 on the negative scale.

**Table 1:** Positive symptoms and negative symptoms of the patient.

Positive symptoms of the patient	Negative symptoms/cognitive symptoms of the patient
Hallucinations: Auditory hallucinations of mother and visual hallucinations of a red phone connecting him to his mother	Affective flattening: Limited range of expressed emotion both at home when found with mother and during hospitalization
Disorganized thinking: Incoherent speech, tangentiality	Alogia/poverty of speech: Demonstrated during hospitalization
Delusions: Fixed belief that he is constantly in communication with his living mother with a red phone that rings overnight	Social withdrawal/isolation: Limited social interaction at home and during hospitalization
	Low energy: Demonstrated during hospitalization

Montreal Cognitive Assessment (MOCA) test revealed a score of 26/30. The patient showed no significant cognitive impairment but had cognitive distortions in understanding the definitions and irreversibility of death. Collateral information provided by his stepbrother revealed that the patient was valedictorian in college before his first psychotic episode. Since then he has never returned to school; he has been cared for by his mother at home. The patient’s bereavement was best categorized to be in the denial stage which was complicated with delusions and auditory hallucinations of a living mother. His treatment plan included the resumption of home medications of clozapine starting at a dose of 50 mg and optimizing to 600 mg over a period of two weeks. In addition, to target his bereavement and distortions relating to his mother’s death, cognitive behavioral therapy (CBT) divided into 20 sessions was scheduled for the course of his hospitalization. CBT treatment usually involves efforts to change faulty thinking patterns, unhelpful behavioral patterns, and help individuals learn to develop coping skills. CBT was aimed at moving him through the stages of grief.

In the first week of hospital admission, the patient demonstrated denial which was modified by the presence of delusions. He was easily approachable and cooperative. However, he displayed poor insight into his reason for hospital admission, stating he had been in the hospital to get his allergies controlled. A gentle exploration of psychotic symptoms was performed given his poor insight and reality testing. He denied any auditory or visual hallucinations as well as suicidal or homicidal intentions at the time. The patient denied feeling sadness, anhedonia, excessive guilt, hopelessness, worthlessness, or a decrease in concentration. The patient did not display any signs of grief during the interview sessions, stating he lives at home with his mother and referring to her in the present tense. The patient’s denial of his mother's death increased as we explored his psychotic delusions. He stated he was expecting a visit from her to the hospital and requested we check the logbook for her signature under an alias name. He did not provide any further information but did provide a telephone number to contact her. The patient stated his mother did not visit as intended but he spoke with her over the phone and she said she would visit him within the next two to three days. He stated, “my mother needs to bring my medication for fatigue before I can go home.” When asked how he had contacted his mother, the patient looked at his palm and stated, “with a gloved telephone that my mother gave me when I was a baby. Can’t you see it? The red phone.” With an excited tone, the patient elaborated on the “gloved telephone” stating it was connected to the main desk and referred to it as a “cord wire.” The patient continued to refer to his mother in the present tense displaying poor insight into his illness and exhibited worsening hallucinations and delusions regarding the “red telephone glove.” The treatment team deferred further exploration of the patient's delusions or hallucinations at this time. 

As we approached the middle of week one, the patient seemed to have regressed in certain aspects of his personality, exhibiting childish hallucinations. The patient elaborated on the “red telephone glove” stating it has “a radar on it, so my mother can track me at any time but I can’t do video chat on it because it would get too hot and burn my hand.” The patient continued to believe his mother was alive, stating she works as a social worker and that he was waiting to speak with her again with the hope that she will sign his medication list. The treatment team seized an opportunity to ask about his understanding of death. He displayed very poor insight into the topic stating he does not believe people die, rather they read books on how to build their muscles and watch television to relax. When asked if he has ever experienced the death of a family member or friend, the patient initially denied this and stated he has never been to a funeral. He later said, “Funerals are a place where people relax their muscles.” When asked about other patients on the unit, the patient stated they were all there for “allergies.” This further displayed his poor insight into his illness. 

The patient had shown some improvement in his mood and affect as well as orientation to person, place, and time as compared to his initial day of hospital admission. The patient was sitting up in bed during his interviews covered in blankets and a towel around his head. This body demeanor remained constant up until the third week of his hospital admission. When asked about his time during college, the patient stated he went to Baruch College and studied “flowers” and later received his Masters in the subject over the span of five years. The patient denied any traumatic or significant event during these years. When asked about his experience as a Valedictorian, based on the collateral information provided by his step-brother, the patient's affect changed. He denied having a step-brother, stating the person who provided that information was a friend who is an alcoholic and should not be trusted. The patient, however, still seemed to be in denial regarding his mother’s death, constantly referring to her in the present tense and awaiting her calls. The patient denied any intimate relationships but enjoyed his time shopping, talking, and eating “eggs and toast” with his mother which they both enjoyed. The patient refused to allow anyone else to contact his mother other than himself to protect her “security.” The patient’s delusions continued to increase regarding his mother, stating he was waiting for his mother to come to be discharged, and that if she is unable to come, he will stay with a babysitter. His discussion on the babysitter was limited; he said it was a private topic. The patient was asked several questions on this day in order to assess fairness, judgment, punishment, reward, and experience. He displayed poor insight on each category, especially in relation to the severity of crimes, fair punishment, and reward. He stated, “The reward depends on the person. It’s private. I like ice cream as a reward”. His responses further proved a childish regression in his understanding of various topics. The patient was asked to assess his current situation of whether or not it was appropriate to treat someone with an allergy on the psychiatric ward versus the medical ward. The patient hesitated at first but eventually said the medical floor was better, however, “The medical floor has the medical tape that grows trees with soaked detergent for the nostrils” perhaps an elaboration of his delusions on the treatment of allergies. 

In the second week of hospital admission, denial intensity decreased and no signs of anger or depression were shown. The patient had a PANSS score of 25 on the positive scale and 32 on the negative scale. CBT was geared towards establishing the moral stage and regression of the patient’s personality. Although the patient admitted to being 50 years old, he often displayed childish delusions. A series of questions were asked in accordance with Kohlberg’s scale to assess the patient’s moral reasoning. The series of questions demonstrated the patient’s poor insight into the idea of punishment and reward. When asked whether or not theft should be punished by law, the patient stated the person who commits the crime or to whom the crime affects, should decide the punishment, as opposed to law enforcement, such as the police or a judge. The patient also expressed his reluctance in helping strangers if they are in need but would help his mother if ever needed. Based on this interview, it was evident that the patient was in the preconventional stages of Kohlberg’s Moral Stage scale, particularly stage 1 (up to age 9). He expressed no insight on law and order and had no concept of punishment; as his responses were expressed in an egocentric viewpoint. 

Throughout week two, CBT was further geared towards assessing the patient’s defense mechanisms and observing his response to the news that his mother will not be coming to see him in the hospital. The patient’s mood during this week can be best described as depressed; the patient stated the reason for this was due to the weather. When confronted that his mother will not pick him up at discharge, nor will she be at home, the patient seemed to accept the idea that his mother is no longer alive. He asserted that he will take care of himself with the help of his mother’s trust fund and babysitters. When pressed to elaborate on this, the patient refused because he considered it a private matter. When asked how he felt not seeing his mother ever again he chuckled and replied, “it’s ok, we went different ways, we have to grow, she has her friends and I have mine.” Despite given unpleasant news, the patient’s reaction was inappropriate lacking proper emotion or reaction. 

Further insight into the patient’s delusions was evident when the interview later shifted to a psychoanalytical point of view. When asked if the patient experienced dreams, he replied “I have a night lamp in my eyes. It helps me sleep. My mother put it there when I was younger. It keeps my mind occupied. It is like a flashlight sticker that was placed in my eye.” The dialogue further proved worsening delusions and insight. The patient seemed to regress back into the denial stage of his mother’s passing at this time. He displayed selective blocking when asked if he could recall the prior conversations, only recalling topics such as his time during college and friends. When confronted about his mother, he stated “yes, she’s divorced/separated now” but did not know from whom, and then eventually he stated he was “ok” with the idea of her passing. 

The patient’s mood and affect seemed brighter as he was seen smiling throughout the interview at the end of week two of his hospital admission. He stated he was waiting for his brother to pick him up once discharged from the hospital. When asked if the patient could recall prior conversations regarding his mother, the patient stated, “Oh yeah, I remember. We talked about the divorce. She’s separated from us now; if she’s feeling better, she’ll be home. She has to have a pulse, she has a pulse now, her pulse is ok, it’s just at work where she has to rest.” In exploring his understanding of death, and after giving him a metaphor of flowers and death, the patient expressed the idea of death to be similar to that of a “wilting flower.” He was able to identify that the conversation mirrored his mother’s death, but he remained adamant on the idea of “divorce” as the reason his mother has “left and moved on.” When asked about his grandmother’s passing, the patient was able to identify her death as no longer having a “pulse” and later stated it was similar to the idea of his mother. This was the first time the patient acknowledged death which contradicted his initial response during week one when he was asked if he had experienced death in a family member. At this stage, we believe the patient began to approach the acceptance stage of bereavement. The patient’s elaboration of death seemed to circulate the idea of reincarnation, stating “people can come back, like reincarnation. Flowers can bring them back and then they will grow.” This demonstrated his understanding of his mother’s death, where he stated that if she wanted to come back in the form of reincarnation she can, however, she will not be present to pick him up from the hospital at the time of discharge. 

During the third week of hospital admission, the patient demonstrated resolution of denial. He elaborated on his understanding of death by exploring the idea of reincarnation and demonstrating his understanding that his mother is no longer present with him. By this time, his PANSS score was 10 on the positive scale and 23 on the negative scale. He remained focused on the hope that his brother will pick him up at discharge and he will go back to living his life with his brother and neighbors, gardening, and playing video games. Upon the conclusion of our CBT, the patient’s final statement when asked who was taking care of his garden, was “whoever my mother left in charge. She died so she can reincarnate herself. When people die, they just don’t come back, they go to dust, ashes to ashes.” This statement signified his acceptance stage in bereavement and his understanding of life after the death of his mother. 

The goal of week four was to evaluate the permanence and effectiveness of the CBT techniques used over the past month. By this time, his PANSS score was 8 on the positive scale and 19 on the negative scale. This week's goal was to examine the overall effect that combined pharmacological and CBT therapy had on the patient’s understanding of his circumstances as well as his level of processing of grief within the accepted bereavement model. As with previous weeks where we were successful in modifying autonomic thought processes, maladaptive core beliefs, and schemas, week four focused on evaluating whether these modifications have aided the patient through the stages of bereavement and processing of the finality of his mother's passing. By establishing a timeline of events with the patient in weeks, we were able to guide him through the events leading up to his admission allowing him the opportunity to re-evaluate previous beliefs held about the weeks prior to admission and the day of admission. Assessment for possibilities of his mother still being alive and present demonstrated that the patient no longer held the same beliefs. He demonstrated an understanding that his mother was no longer alive and that her remains were in the form of ashes in a mantle. A family meeting was held with his stepbrother and arrangement for his mother’s funeral was discussed. He participated in the funeral arrangement discussions and made arrangements to attend the funeral.

## Discussion

The Kubler-Ross model analyzes the emotional processing of death and the linear thinking that patients experience when having to deal with grief and the concept of death. The model recognizes five stages that linearly follow one after the other. The initial and most common emotional response to the knowledge of impending death is denial. It is seen as a defense mechanism that helps ease anxiety and fearful thoughts, allowing one to come to terms with the concept of death on their own until they are ready to cope constructively. The second stage is anger; once a patient comes to terms with what is happening, he or she may become very angry. Feelings of rage or resentment may overcome this person and the anger may be directed at others as well. The third stage is bargaining and it involves the hope that the individual can avoid a cause of grief by giving something in return. The patient may try to bargain with God, doctors, or family. The fourth stage is depression. At this point, the patient realizes that there is nothing left to do after denial, anger, and bargaining have failed. In this state, he or she may become silent, refuse visitors, and spend much of the time mournful and sullen. The last stage is acceptance in which the patient embraces his or her future, which typically comes with a calm, retrospective view for the individual, and a stable condition of emotions [1]. 

The Kubler-Ross model was used on our patient but given the psychiatric mental condition of the individual, the emotional processing and linearity of thought are different compared to that of a patient without a mental health history (Table 2). In week one, the patient was in a denial stage. He was in denial of his mother’s death and the concept of death in general. The patient exhibited delusions and hallucinations and his cognitive distortions represented his level of understanding of the irreversibility of death. In week two, the patient was in a brief depressed stage which later led to the acceptance stage as he accepted that his mother was no longer with him. While he grasped a better understanding of the situation, he still did not come to accept the concept of death as a whole. During week three, the patient understood the meaning of death in that his mother was never coming back. This approach was effective in our patient and could be used as a model for treating and understanding bereavement in patients with schizophrenia.

**Table 2:** Comparing Kubler-Ross Model of bereavement with the Modified Kubler-Ross Model.

Kubler-Ross Model	Modified Kubler-Ross Model
Denial: The first reaction is denial. In this stage, individuals believe the death is somehow mistaken, and cling to a false, preferable reality.	Denial with perceptual disturbances: There is denial, however, the degree in denial is modified by the presence of hallucinations and delusions congruent with the denial. Auditory hallucinations of the dead, visual hallucinations of objects associated with the dead may be present and be severe enough to need treatment, in addition to helping the patient move through denial.
Anger: When the individual recognizes that denial cannot continue, they become frustrated and express anger towards the situation.	Anger blunted by negative symptoms: Patients may have negative symptoms including blunting of affect and apathy that prevent the expression of frustration. Therefore, this stage may not be readily apparent in patients with schizophrenia.
Bargaining: The patient attempt to postpone their sadness by imagining “what if” scenarios.	Bargaining limited by thought disorder and delusional beliefs: The presence of disorganization of thought may make the creation of clear “what if” scenarios difficult in patients with Schizophrenia. Therefore, this stage may not be present. If present, the "what if" scenarios may involve scenarios with persecutory content, or scenarios that demonstrate magical thinking in patients who exhibit delusional thought,
Depression: The individual despairs at the recognition of the situation. In this state, the individual may become silent, refuse visitors, and spend much of the time mournful and sullen.	Depression: In patients with schizophrenia, the presence of negative symptoms such as apathy and affective blunting make this stage not apparent or not exiting in patients with schizophrenia.
Acceptance: Individuals embrace the tragic event and a more stable condition of emotions	Acceptance: This is the stage in which patients with schizophrenia, embrace the tragic event. Given affective blunting limiting emotional expression, emotional stability may not be a sign of this stage. A sign of acceptance will be resolution of denial and accompanying psychotic symptoms such as perceptual hallucinations and delusions.

Table 2 compares the Kubler-Ross Model with the Modified Version of Kubler-Ross. In the modified version of Kubler-Ross Model, there is a predominance of the denial stage and acceptance stage, with the anger and depression stage being limited by negative symptoms, and bargaining stage limited by thought disorder and delusional beliefs. The most apparent progression of the patient from an objective point of view may therefore be from denial with perceptual disturbances and delusions, to bargaining with magical thinking and thought disorder, to acceptance with resolution of denial and psychotic symptoms. As seen in our patient. The approach needed is a combination of CBT and medications.

The defense mechanisms we observed to be present in our patient include the following: avoidance, conversion, isolation, denial, rationalization, repression, and regression. The patient displayed avoidance when asked about topics such as the “babysitters”, but displayed conversion on all encounters when stating he is “under the weather” and “covered in blankets” on some instances as a justification of his anxiety. The patient expressed denial of his mother’s death; his isolative defense mechanism became apparent as he created an alternate reality with the “gloved telephone” and his mother’s visits. The patient showed repression about the death of his mother in their apartment. Based on Kohlberg’s moral stage the patient’s cognitive state has regressed to a preconventional stage 1 (up to nine years old). 

A modified model of Kubler-Ross was used for the patient, but currently, there are different models in grief counseling, such as the recognition of the uniqueness of the griever, questioning the grief work hypothesis, continuing bonds with the deceased, and recognition of culture. “The recognition of the uniqueness of the griever” takes into consideration the five stages of Kubler-Ross, but the older models implicate a universality and logical progression that ignore the complexity and individuality of the grieving experience. Every individual is influenced by various factors such as culture, personality, and experiences. Therefore a grief counselor should always take into consideration these aspects when assessing a patient. “Questioning the grief work hypothesis” takes into consideration the “grief work” concept for some individuals (originally elaborated by Freud, who described this concept as a period of working through thoughts, memories, and emotions associated with the loss), but there is the notion that not every patient can benefit from it, although, some individuals cope better by using methods of distraction or suppression of emotions. “Continuing bonds with the deceased” is a model that states that severing ties with the deceased is not necessarily the best way to achieve the resolution of loss. There are four ways in which many bereaved individuals maintain bonds with the dead: by sensing the presence of the dead, by talking to that person, by using the deceased as a moral guide, and by talking about that person to someone else. “Recognition of culture” states that it is important that counselors identify clients’ unique cultural influences and their impact on the grieving process rather than working from stereotypes [5]. 

Literature on bereavement in mental illness such as schizophrenia are not extensive. A study of bereavement in the patients with serious mental illness [6] examined the role of situational factors in grieving persons who are diagnosed with serious mental illness. The situational factors found to be predictors of severe and prolonged grief were: residing with a close friend or family member at the time of the death, the suddenness of death, having low social support, and having concurrent stressors [6]. Concurrent stressors that can intensify grief or lengthen the time of grief include the concomitant loss of health or financial security, forced relocation, the sacrifice of a job to care for the dying person, or the burden of dealing with legal and practical problems related to the death. Macias et al. in a study of 148 individuals with serious mental illness, 22% reported the death of a close friend or family member as a significant life event that resulted in relatively acute and brief grief (10%) or severe and prolonged grief (12%). This study did not take into consideration a pharmacological approach or CBT for these patients but suggests offering practical planning for bereavement as an essential service, such as counseling, helping with funeral arrangements, financial planning, or arranging for a move to supported housing [6]. 

It may be suggested to focus on the depressive symptoms in patients with schizophrenia who are grieving. A study on elderly patients with schizophrenia and depression [7] did not examine grief and how to cope with it in a schizophrenic patient, but it observed the effects of citalopram in patients presenting with schizophrenia and depression already taking second-generation antipsychotic drugs. Citalopram was started at 20 mg daily and then either decreased to 10 mg daily or increased to 40 mg daily. All of the patients in the study improved with citalopram and had mild side effects or adverse events [7]. In focusing on depression as an indicator of bereavement, however, especially in the setting of life events, one needs to take into account the changes in emotional expression in patients with schizophrenia [8]. A study showed that schizophrenic patients have a deficit in the expressive component but not in the physiological components of emotion, showing that they can experience a bereavement process [8]. 

We can make a comparison between our patient and a case report of a 50-year-female diagnosed with paranoid schizophrenia and anxiety disorder who also went through bereavement for the loss of her mother [9]. In this case, the Kubler-Ross model was not used, so we cannot assess if she went through the five stages. However combining CBT, psychodynamic therapy, and psychopharmacology proved to be very useful and to lead to the recovery of the patient [9]. The case had one similarity with our patient presented above, which was the use of delusions to try to escape the fact that their mothers had died [9]. 

These studies all provide evidence of the complex nature of bereavement in patients with schizophrenia. The need for research becomes clear from the paucity of literature directly dealing with the subject.

## Conclusions

There are limited studies on bereavement and managing bereavement in patients with mental illness. A modified model of Kubler-Ross that takes into account abnormal thought content and processes in schizophrenia may be utilized to model and treat bereavement in schizophrenia patients. More studies need to be done on the possible utilization of our modified model and therapeutic approach in the treatment of schizophrenia and bereavement.
